# Supplementary material for: Longitudinal deep multi-omics profiling in a CLN3Δex7/8 minipig model identifies biomarker signatures of disease
Source: Commun Med (Lond). 2026 Mar 3;6:132. doi: 10.1038/s43856-025-01227-5 (PMC12957377; doi:10.1038/s43856-025-01227-5)
Supplement: Supplementary file 2 — Supplementary Information [file 43856_2025_1227_MOESM2_ESM.pdf]

## Supplementary Figures

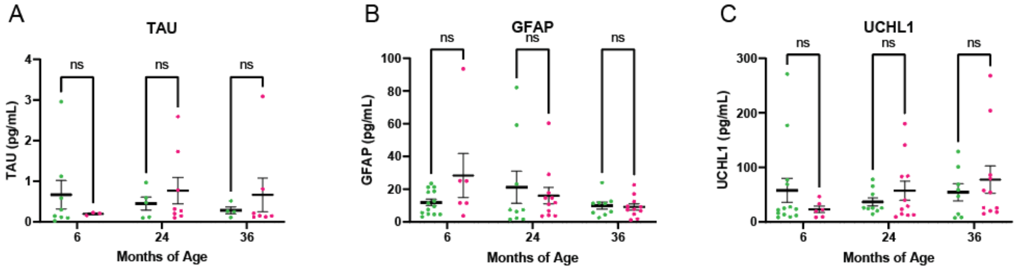


**Supplementary Figure 1: Neurology 4-PlexA Targeted Proteomic Analysis.** The total measurement of (A) TAU, (B) glial fibrillary acidic protein (GFAP), (C) and ubiquitin carboxyl-terminal hydrolase L1 (UCHL1) in wildtype *CLN3*$^{\Delta ex7/8}$ minipig serum at 6-, 24- and 36-months. No significant differences were observed. Two-way ANOVA with Šidák correction for multiple comparisons, 95% confidence interval, n = 6; 9; 9 animals respectively, mean +/- SEM.

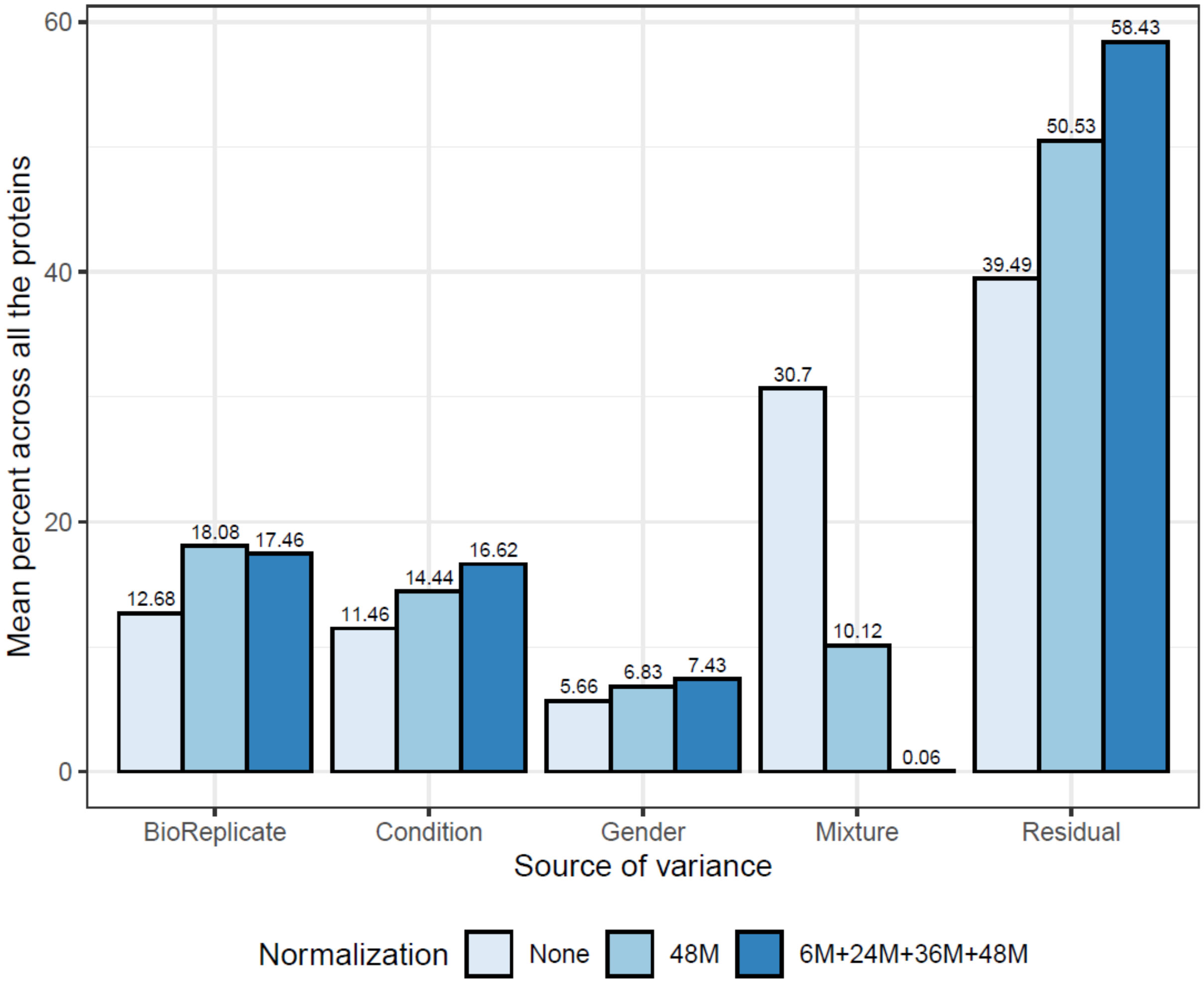


**Supplementary Figure 2: Variance Components Analysis on the Protein Intensities Generated by Different Normalization Methods.** In the context of normalization methods, "None" indicates no normalization was performed. "48M" refers to the usage of pooling samples from 48-month-old subjects as the reference channel, while "6M+24M+36M+48M" signifies the utilization of the mean across all samples from all ages as the artificial reference channel. The x-axis represents the sources of different variance components, with "Conditions" representing the combination of age and genotype, and "BioReplicate" representing distinct pigs. The percentage of each variance component in the overall variation was calculated for each protein. The y-axis displays the average percentage of each variance component across all proteins. Utilizing normalization based on all the samples effectively mitigates the between-mixture variance, eliminating the undesirable batch effect.

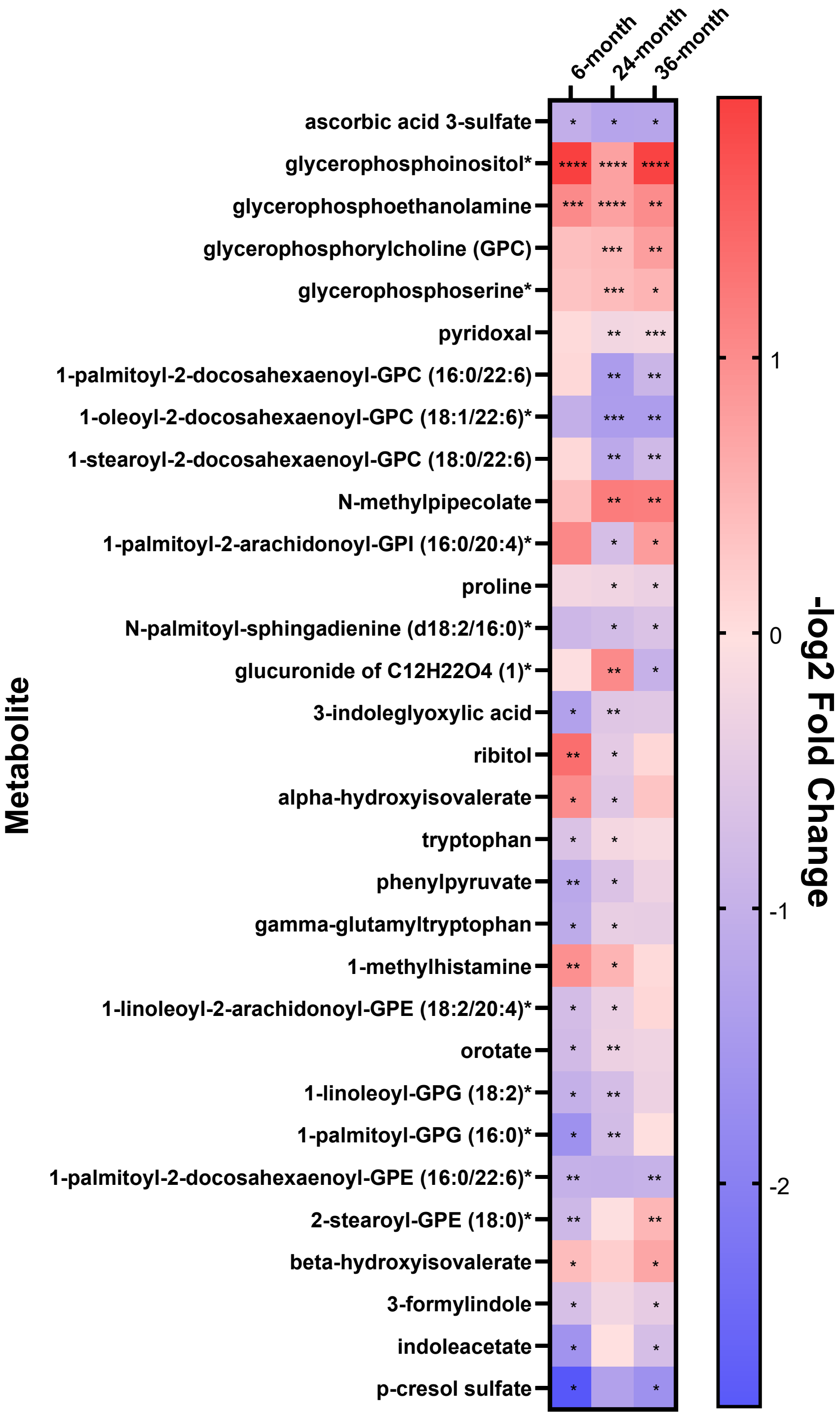


**Supplementary Figure 3: Differentially expressed metabolites shared by two or more timepoints.** Significantly dysregulated metabolite species shared by two or more time points. Student's two-tailed t-test. N = 6; 9; 9 animals respectively. Corresponding p-values can be found in Supplemental Data 3.

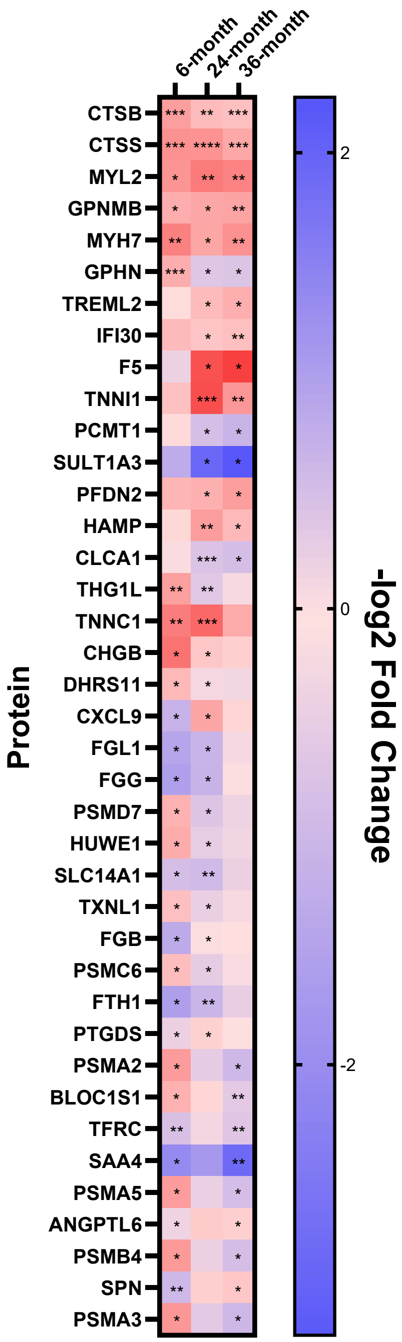


**Supplementary Figure 4: Differentially expressed proteins shared by two or more timepoints.** Significantly dysregulated protein species shared by two or more time points. Student's two-tailed t-test. N = 6; 9; 9 animals respectively. Corresponding p-values can be found in Supplemental Data 4. All abbreviated protein names reflect official gene names under Human Genome Organization (HUGO).

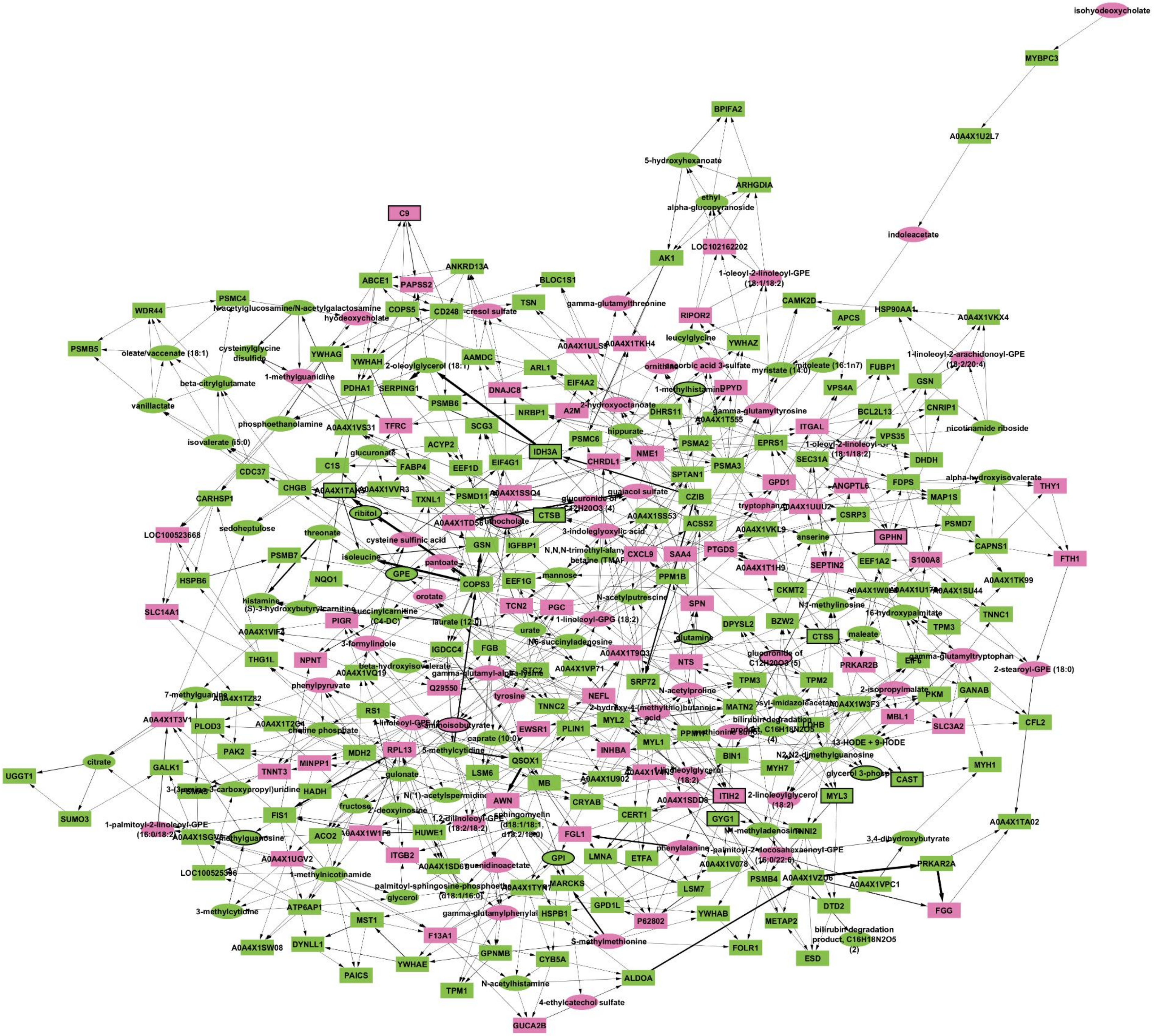

isohyodeoxycholate
MYBPC3
A0A4X1U2L7
BPIFA2
5-hydroxyhexanoate
ARHGDIA
ethyl
alpha-glucopyranoside
C9
indoleacetate
LOC102162202
AK1
1-oleoyl-2-linoleoyl-GPE
(18:1/18:2)
ANKRD13A
ABCE1
PAPSS2
BLOC1S1
PSMC4
CAMK2D
TSN
CD248
COPS5
gamma-glutamylthreonine
APCS
HSP90AA1
A0A4X1VKX4
WDR44
N-acetylglucosamine/N-acetylgalactosamine
hyodeoxycholate
RIPOR2
leucylglycine
A0A4X1ULS5
A0A4X1TKH4
YWHAZ
PSMB5
oleate/vaccenate (18:1)
cysteinylglycine
disulfide
YWHAG
YWHAH
AAMDC
ornithine
myristate (14:0)
FUBP1
1-linoleoyl-2-arachidonoyl-GPE
(18:2/20:4)
beta-citrylglutamate
1-methylguanidine
2-oleoylglycerol (18:1)
ARL1
EIF4A2
DNAJC8
1-methylhistamine
DPYD
VPS4A
GSN
PDHA1
SERPING1
PSMB6
vanillactate
2-hydroxyoctanoate
A2M
NRBP1
DHRS11
gamma-glutamyltyrosine
BCL2L13
CNRIP1
phosphoethanolamine
A0A4X1VS31
TFRC
SCG3
A0A4X1T555
nicotinamide riboside
hippurate
ITGAL
VPS35
isovalerate (i5:0)
ACYP2
IDH3A
PSMC6
PSMA2
EPRS1
glucuronate
1-oleoyl-2-linoleoyl-GPC
(18:1/18:2)
C1S
EIF4G1
CHRDL1
NME1
CDC37
FABP4
EEF1D
SPTAN1
PSMA3
SEC31A
DHDH
alpha-hydroxyisovalerate
CHGB
A0A4X1TAK5
A0A4X1VVR3
CARHSP1
A0A4X1SSQ4
guaiacol sulfate
GPD1
ANGPTL6
FDPS
THY1
ribitol
TXNL1
PSMD11
glucuronide of
C12H20O3 (4)
CZIB
tryptophan
A0A4X1UUU2
MAP1S
CTSB
CSRP3
A0A4X1TD56
lithocholate
3-indoleglyoxylic acid
A0A4X1SS53
LOC100523668
sedoheptulose
threonate
ACSS2
A0A4X1VKL9
PSMD7
cysteine sulfinic acid
anserine
GPHN
GSN
IGFBP1
N,N,N-trimethyl-alanylproline
betaine (TMAP)
isoleucine
CXCL9
SAA4
PTGDS
CAPNS1
PSMB7
pantoate
SEPTIN2
S100A8
EEF1A2
FTH1
A0A4X1T1H9
GPE
mannose
HSPB6
NQO1
COPS3
EEF1G
PPM1B
A0A4X1TK99
CKMT2
A0A4X1W0B8
A0A4X1U17
A0A4X1SU44
histamine
(S)-3-hydroxybutyrylcarnitine
orotate
TCN2
PGC
N-acetylputrescine
SPN
N1-methylinosine
TPM3
SLC14A1
succinylcarnitine
(C4-DC)
laurate (12:0)
16-hydroxypalmitate
TNNC1
1-linoleoyl-GPG (18:2)
DPYSL2
BZW2
PIGR
A0A4X1VIF4
urate
glutamine
CTSS
maleate
3-formylindole
N6-succinyladenosine
glucuronide of
C12H20O3 (5)
THG1L
A0A4X1T9Q3
NPNT
IGDCC4
FGB
NTS
PRKAR2B
EIF6
gamma-glutamyltryptophan
2-stearoyl-GPE (18:0)
beta-hydroxyisovalerate
A0A4X1VQ19
STC2
A0A4X1VP71
gamma-glutamyl-alpha-lysine
SRP72
N-acetylproline
TPM3
TPM2
phenylpyruvate
2-isopropylmalate
PKM
GANAB
7-methylguanine
A0A4X1TZ82
Q29550
tyrosine
NEFL
MATN2
A0A4X1W3F3
TNNC2
2-hydroxy-4-(methylthio)butanoic
acid
MBL1
A0A4X1T3V1
RS1
A0A4X1T2C4
1-linoleoyl-GPE
3-aminoisobutyrate
MYL2
bilirubin degradation
product, C16H18N2O5
(4)
LDHB
SLC3A2
CFL2
PLOD3
choline phosphate
caprate (10:0)
EWSR1
PLIN1
13-HODE + 9-HODE
PAK2
MDH2
RPL13
5-methylcytidine
INHBA
MYL1
BIN1
N2,N2-dimethylguanosine
citrate
MINPP1
TNNT3
QSOX1
MYH7
MYH1
UGGT1
GALK1
gulonate
glycerol 3-phosphate
CAST
HADH
LSM6
N('1)-acetylspermidine
MB
A0A4X1U902
2-linoleoylglycerol
(18:2)
ITIH2
MYL3
fructose
2'-deoxyinosine
AWN
CRYAB
A0A4X1SDD6
FIS1
1,2-dilinoleoyl-GPC
(18:2/18:2)
sphingomyelin
(d18:1/18:1,
d18:2/18:0)
CERT1
GYG1
A0A4X1TA02
SUMO3
1-palmitoyl-2-linoleoyl-GPE
(16:0/18:2)
A0A4X1SGV5
methylguanosine
ACO2
A0A4X1W1F8
HUWE1
FGL1
N1-methyladenosine
TNNI2
3,4-dihydroxybutyrate
A0A4X1UGV2
ITGB2
phenylalanine
1-palmitoyl-2-docosahexaenoyl-GPE
(16:0/22:6)
A0A4X1SD67
guanidinoacetate
GPI
LMNA
ETFA
A0A4X1V078
PRKAR2A
LOC100525396
1-methylnicotinamide
MARCKS
PSMB4
A0A4X1VZ06
A0A4X1VPC1
palmitoyl-sphingosine-phosphoethanolamine
(d18:1/16:0)
A0A4X1TYR7
glycerol
LSM7
DTD2
FGG
3-methylcytidine
ATP6AP1
MST1
gamma-glutamylphenylalanine
HSPB1
GPD1L
P62802
YWHAB
METAP2
bilirubin degradation
product, C16H18N2O5
(2)
A0A4X1SW08
DYNLL1
F13A1
S-methylmethionine
FOLR1
YWHAE
GPNMB
CYB5A
ESD
PAICS
TPM1
N-acetylhistamine
ALDOA
4-ethylcatechol sulfate
GUCA2B

**Supplementary Figure 5: Bayesian Hill Climbing inference algorithm with Bayesian Dirichlet Equivalent (BDe) of all dysregulated analytes at 6-months.** Significance calculated by uncorrected Student's t-test, n=6, p-value < 0.5. Eclipses represent metabolites, rectangles represent proteins. Green: Upregulated, Magenta: Downregulated. Outlined analytes are in top 10 list of proteins or metabolites. Edge width corresponds to BDe score (reflective of the probability of the relationship between two analytes). Corresponding  p-values and source data can be found in Supplementary Data 7. All abbreviated protein names reflect official gene names under Human Genome Organization (HUGO).

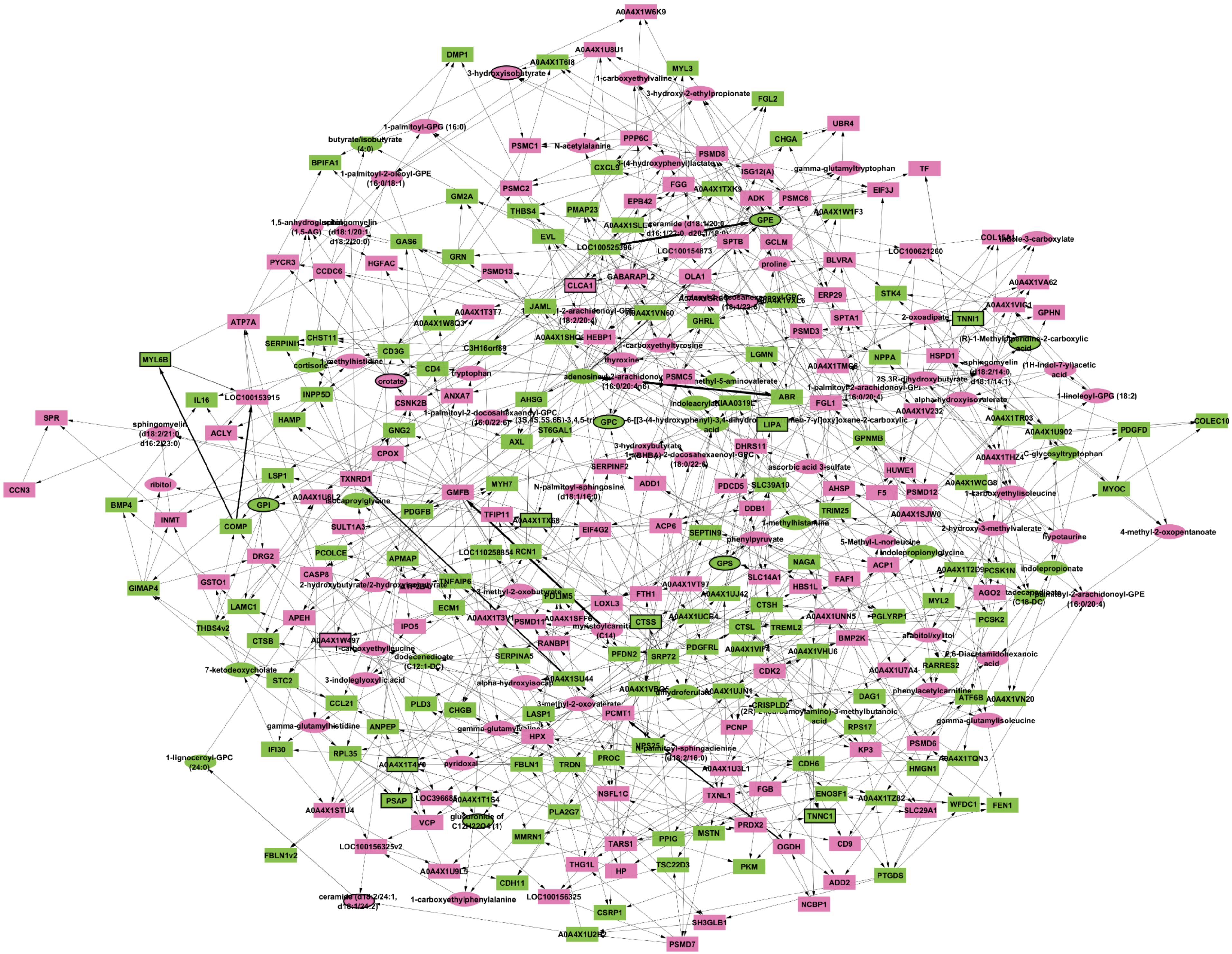

A0A4X1W6K9
A0A4X1U8U1
DMP1
A0A4X1T6I8
MYL3
3-hydroxyisobutyrate
1-carboxyethylvaline
3-hydroxy-2-ethylpropionate
FGL2
UBR4
1-palmitoyl-GPG (16:0)
butyrate/isobutyrate (4:0)
PSMC1
N-acetylalanine
PPP6C
CHGA
PSMD8
BPIFA1
1-palmitoyl-2-oleoyl-GPE (16:0/18:1)
CXCL9
3-(4-hydroxyphenyl)lactate
ISG12(A)
gamma-glutamyltryptophan
TF
PSMC2
FGG
A0A4X1TXK9
EIF3J
GM2A
EPB42
ADK
PSMC6
THBS4
PMAP23
A0A4X1W1F3
A0A4X1SLE4
GPE
GAS6
EVL
SPTB
GCLM
COL1A2
indole-3-carboxylate
PYCR3
LOC100525396
LOC100154873
LOC100621260
HGFAC
GRN
proline
BLVRA
CCDC6
PSMD13
CLCA1
GABARAPL2
OLA1
A0A4X1VA62
STK4
ERP29
JAML
A0A4X1T3T7
A0A4X1VN60
GHRL
2-oxoadipate
TNNI1
GPHN
ATP7A
A0A4X1W8Q3
SPTA1
A0A4X1VIG1
PSMD3
CHST11
SERPINI1
A0A4X1SHQ8
HEBP1
1-carboxyethyltyrosine
(R)-1-Methylpiperidine-2-carboxylic acid
MYL6B
CD3G
C3H16orf89
LGMN
HSPD1
NPPA
thyroxine
cortisone
1-methylhistidine
CD4
tryptophan
A0A4X1TMG6
sphingomyelin (d18:2/14:0, d18:1/14:1)
(1H-indol-7-yl)acetic acid
orotate
PSMC5
2S,3R-dihydroxybutyrate
INPP5D
ANXA7
AHSG
ABR
1-linoleoyl-GPG (18:2)
LOC100153915
CSNK2B
FGL1
alpha-hydroxyisovalerate
IL16
indoleacrylate
KIAA0319L
A0A4X1V232
SPR
HAMP
1-palmitoyl-2-docosahexaenoyl-GPC (16:0/22:6)
GPC
LIPA
A0A4X1TR03
COLEC10
sphingomyelin (d18:2/21:0, d16:2/23:0)
ACLY
GNG2
ST6GAL1
A0A4X1U902
PDGFD
AXL
GPNMB
CPOX
3-hydroxybutyrate (BHBA)
DHRS11
A0A4X1THZ4
C-glycosyltryptophan
SERPINF2
ascorbic acid 3-sulfate
HUWE1
LSP1
TXNRD1
ADD1
PDCD5
SLC39A10
ribitol
MYH7
GMFB
N-palmitoyl-sphingosine (d18:1/16:0)
AHSP
F5
PSMD12
A0A4X1WCG8
MYOC
CCN3
A0A4X1U6L2
isocaproylglycine
1-carboxyethylisoleucine
BMP4
GPI
PDGFB
TFIP11
DDB1
TRIM25
A0A4X1SJW0
INMT
COMP
SULT1A3
A0A4X1TX68
EIF4G2
ACP6
1-methylhistamine
SEPTIN9
2-hydroxy-3-methylvalerate
hypotaurine
4-methyl-2-oxopentanoate
phenylpyruvate
5-Methyl-L-norleucine
PCOLCE
LOC110258854
RCN1
indolepropionylglycine
DRG2
APMAP
GPS
NAGA
ACP1
A0A4X1T2D9
PCSK1N
indolepropionate
CASP8
SLC14A1
GIMAP4
GSTO1
2-hydroxybutyrate/2-hydroxyisobutyrate
TNFAIP6
3-methyl-2-oxobutyrate
FTH1
A0A4X1VT97
FAF1
HBS1L
AGO2
PDLIM5
A0A4X1UJ42
MYL2
LOXL3
LAMC1
ECM1
CTSH
1-palmitoyl-2-arachidonoyl-GPE (16:0/20:4)
APEH
A0A4X1T3Y1
PSMD11
A0A4X1SFF6
CTSS
A0A4X1UCB4
A0A4X1UNN5
PGLYRP1
PCSK2
IPO5
THBS4v2
myristoylcarnitine (C14)
CTSL
TREML2
BMP2K
CTSB
A0A4X1W497
RANBP1
arabitol/xylitol
PDGFRL
A0A4X1VIF4
1-carboxyethylleucine
PFDN2
SRP72
A0A4X1VHU6
2,6-Diacetamidohexanoic acid
SERPINA5
dodecenedioate (C12:1-DC)
7-ketodeoxycholate
RARRES2
CDK2
A0A4X1U7A4
3-indoleglyoxylic acid
STC2
alpha-hydroxyisocaproate
A0A4X1SU44
phenylacetylcarnitine
A0A4X1VB06
dihydroferulate
A0A4X1UJN1
ATF6B
A0A4X1VN20
DAG1
CCL21
PLD3
3-methyl-2-oxovalerate
CRISPLD2
CHGB
LASP1
PCMT1
gamma-glutamylisoleucine
gamma-glutamylhistidine
ANPEP
gamma-glutamylvaline
PCNP
RPS17
HPX
PSMD6
IFI30
RPL35
N-palmitoyl-sphingadienine (d18:2/16:0)
KP3
A0A4X1TQN3
1-lignoceroyl-GPC (24:0)
A0A4X1T4V0
pyridoxal
FBLN1
TRDN
PROC
CDH6
HMGN1
A0A4X1U3L1
FGB
PSAP
LOC396685
A0A4X1T1S4
NSFL1C
ENOSF1
A0A4X1TZ82
WFDC1
FEN1
A0A4X1STU4
TXNL1
TNNC1
SLC29A1
VCP
glucuronide of C12H22O4 (1)
PLA2G7
PRDX2
MMRN1
MSTN
PPIG
FBLN1v2
TARS1
OGDH
CD9
LOC100156325v2
PTGDS
THG1L
TSC22D3
A0A4X1U9L5
HP
PKM
ADD2
CDH11
ceramide (d18:2/24:1, d18:1/24:2)
1-carboxyethylphenylalanine
LOC100156325
NCBP1
CSRP1
SH3GLB1
A0A4X1U2F2
PSMD7

**Supplementary Figure 6: Bayesian Hill Climbing inference algorithm with Bayesian Dirichlet Equivalent (BDe) of all dysregulated analytes at 24-months.** Significance calculated by uncorrected Student's t-test, n=9, p-value < 0.5. Eclipses represent metabolites, rectangles represent proteins. Green: Upregulated, Magenta: Downregulated. Outlined analytes are in top 10 list of proteins or metabolites. Edge width corresponds to BDe score (reflective of the probability of the relationship between two analytes. Corresponding p-values and source data can be found in Supplementary Data 8. All abbreviated protein names reflect official gene names under Human Genome Organization (HUGO).

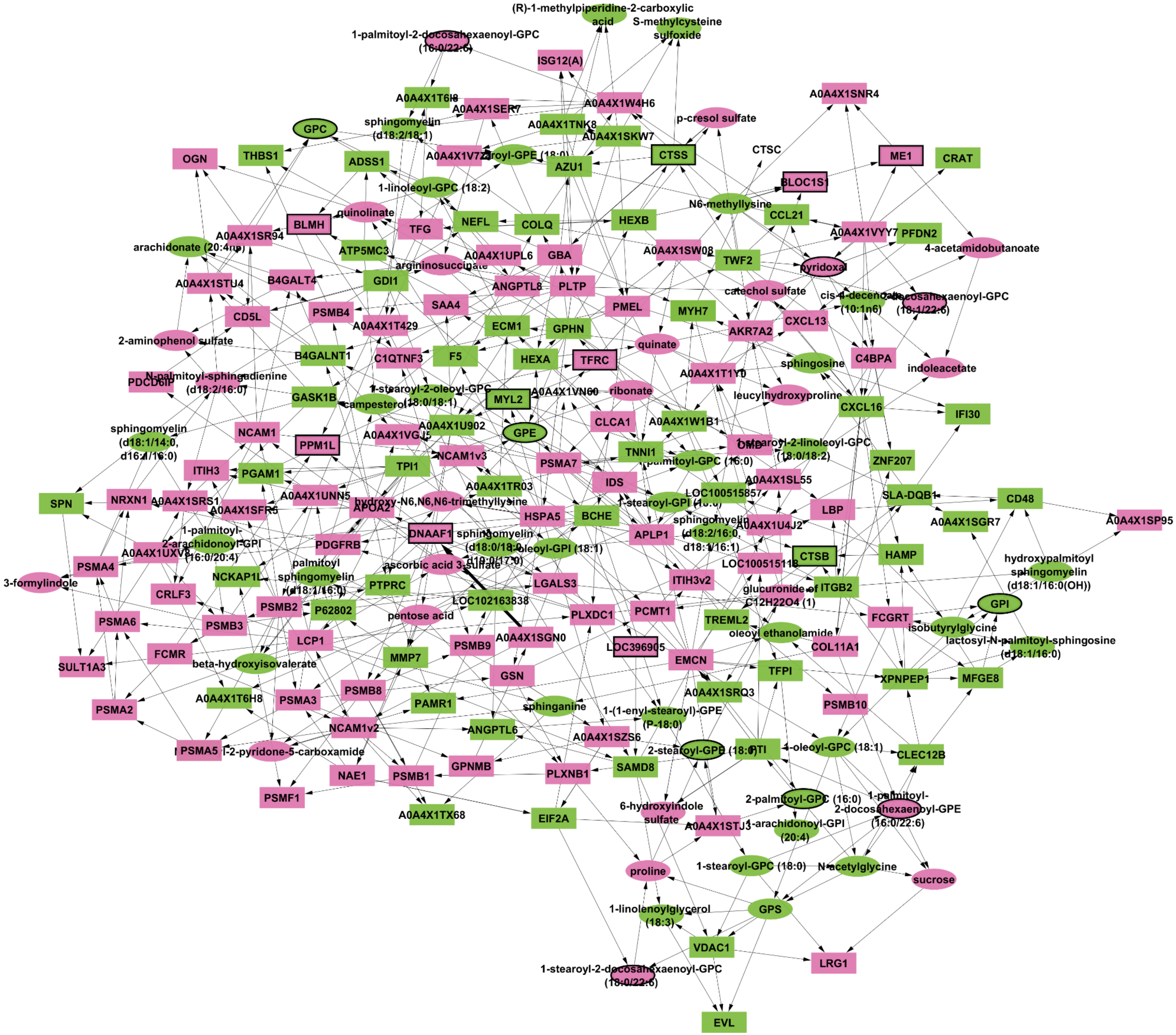
(R)-1-methylpiperidine-2-carboxylic acid
S-methylcysteine sulfoxide
1-palmitoyl-2-docosahexaenoyl-GPC (16:0/22:6)
ISG12(A)
A0A4X1T6I8
A0A4X1SER7
A0A4X1W4H6
A0A4X1SNR4
GPC
sphingomyelin (d18:2/18:1)
A0A4X1TNK8
p-cresol sulfate
A0A4X1SKW7
OGN
THBS1
ADSS1
A0A4X1V7Z2
stearoyl-GPE (18:0)
AZU1
CTSS
CTSC
ME1
CRAT
BLOC1S1
1-linoleoyl-GPC (18:2)
N6-methyllysine
CCL21
quinolinate
NEFL
COLQ
HEXB
A0A4X1VYY7
PFDN2
BLMH
TFG
A0A4X1SR94
arachidonate (20:4n6)
ATP5MC3
A0A4X1UPL6
GBA
A0A4X1SW08
TWF2
4-acetamidobutanoate
argininosuccinate
pyridoxal
A0A4X1STU4
B4GALT4
GDI1
ANGPTL8
PLTP
catechol sulfate
cis-4-decenoate (10:1n6)
1-docosahexaenoyl-GPC (18:1/22:6)
CD5L
PSMB4
SAA4
PMEL
MYH7
CXCL13
A0A4X1T429
ECM1
GPHN
AKR7A2
2-aminophenol sulfate
quinate
C4BPA
B4GALNT1
C1QTNF3
F5
HEXA
TFRC
sphingosine
indoleacetate
PDCD6IP
N-palmitoyl-sphingadienine (d18:2/16:0)
A0A4X1T1Y0
1-stearoyl-2-oleoyl-GPC (18:0/18:1)
GASK1B
MYL2
A0A4X1VN60
ribonate
leucylhydroxyproline
campesterol
CXCL16
IFI30
A0A4X1U902
CLCA1
A0A4X1W1B1
sphingomyelin (d18:1/14:0, d16:1/16:0)
NCAM1
A0A4X1VGJ5
GPE
PPM1L
OMD
1-stearoyl-2-linoleoyl-GPC (18:0/18:2)
NCAM1v3
TNNI1
PSMA7
1-palmitoyl-GPC (16:0)
ZNF207
ITIH3
PGAM1
TPI1
IDS
A0A4X1SL55
A0A4X1TR03
SLA-DQB1
CD48
SPN
NRXN1
A0A4X1SRS1
A0A4X1UNN5
LOC100515857
hydroxy-N6,N6,N6-trimethyllysine
1-stearoyl-GPI (18:0)
LBP
A0A4X1SFR5
APOA2
HSPA5
BCHE
A0A4X1SGR7
A0A4X1SP95
sphingomyelin (d18:2/16:0, d18:1/16:1)
A0A4X1U4J2
1-palmitoyl-2-arachidonoyl-GPI (16:0/20:4)
DNAAF1
APLP1
A0A4X1UXV8
PDGFRB
sphingomyelin (d18:0/18:0, d19:0/17:0)
1-oleoyl-GPI (18:1)
CTSB
HAMP
PSMA4
palmitoyl sphingomyelin (d18:1/16:0)
ascorbic acid 3-sulfate
LOC100515118
hydroxypalmitoyl sphingomyelin (d18:1/16:0(OH))
NCKAP1L
3-formylindole
PTPRC
LGALS3
ITIH3v2
ITGB2
CRLF3
glucuronide of C12H22O4 (1)
GPI
LOC102163838
PSMB2
P62802
PCMT1
PSMA6
pentose acid
PLXDC1
TREML2
FCGRT
PSMB3
isobutyrylglycine
LCP1
A0A4X1SGN0
oleoyl ethanolamide
PSMB9
LOC396905
lactosyl-N-palmitoyl-sphingosine (d18:1/16:0)
FCMR
COL11A1
beta-hydroxyisovalerate
MMP7
EMCN
SULT1A3
TFPI
GSN
XPNPEP1
MFGE8
A0A4X1SRQ3
A0A4X1T6H8
PSMB8
PSMA3
PAMR1
PSMB10
PSMA2
sphinganine
1-(1-enyl-stearoyl)-GPE (P-18:0)
NCAM1v2
ANGPTL6
A0A4X1SZS6
PSMA5
1-methyl-2-pyridone-5-carboxamide
2-stearoyl-GPE (18:0)
PTI
1-oleoyl-GPC (18:1)
CLEC12B
GPNMB
NAE1
PSMB1
PLXNB1
SAMD8
2-palmitoyl-GPC (16:0)
1-palmitoyl-2-docosahexaenoyl-GPE (16:0/22:6)
PSMF1
A0A4X1TX68
EIF2A
6-hydroxyindole sulfate
A0A4X1STJ3
1-arachidonoyl-GPI (20:4)
proline
1-stearoyl-GPC (18:0)
N-acetylglycine
sucrose
1-linolenoylglycerol (18:3)
GPS
VDAC1
LRG1
1-stearoyl-2-docosahexaenoyl-GPC (18:0/22:6)
EVL

**Supplementary Figure 7: Bayesian Hill Climbing inference algorithm with Bayesian Dirichlet Equivalent (BDe) of all dysregulated analytes at 36-months.** Significance calculated by uncorrected Student's t-test, n=9, p-value < 0.5. Eclipses represent metabolites, rectangles represent proteins. Green: Upregulated, Magenta: Downregulated. Outlined analytes are in top 10 list of proteins or metabolites. Edge width corresponds to BDe score (reflective of the probability of the relationship between two analytes). Corresponding p-values and source data can be found in Supplementary Data 9. All abbreviated protein names reflect official gene names under Human Genome Organization (HUGO).

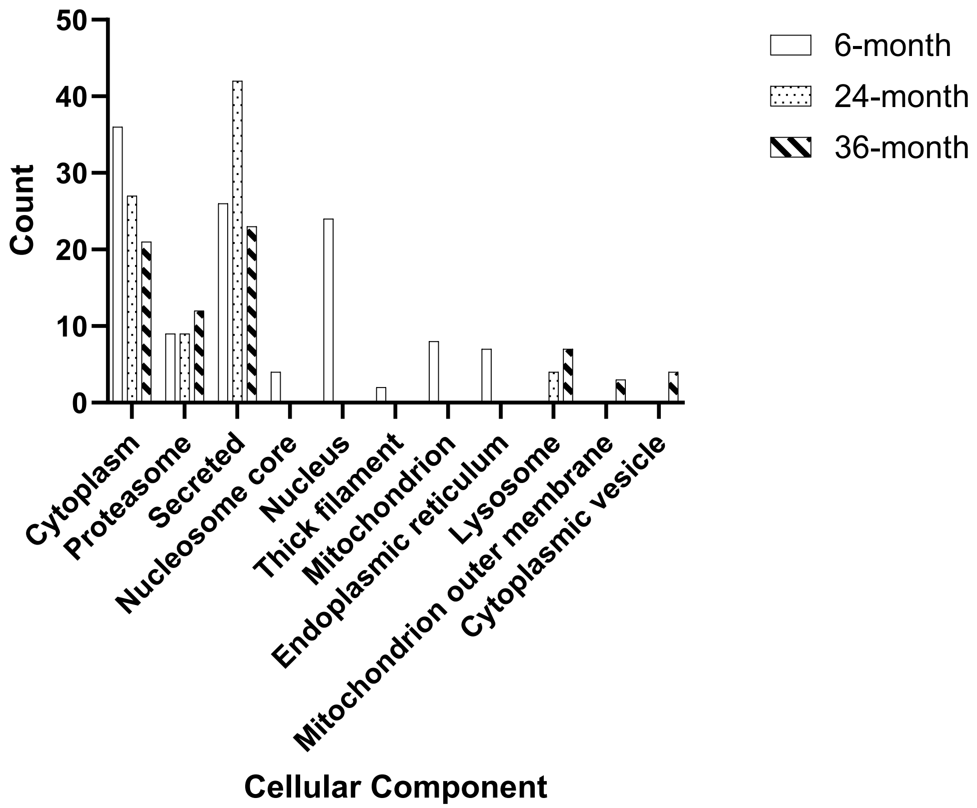


**Supplementary Figure 8:** Cellular components associated with genotype at 6-, 24-, and 36-months using the DAVID functional annotation tool. N=6,9,9, respectively.

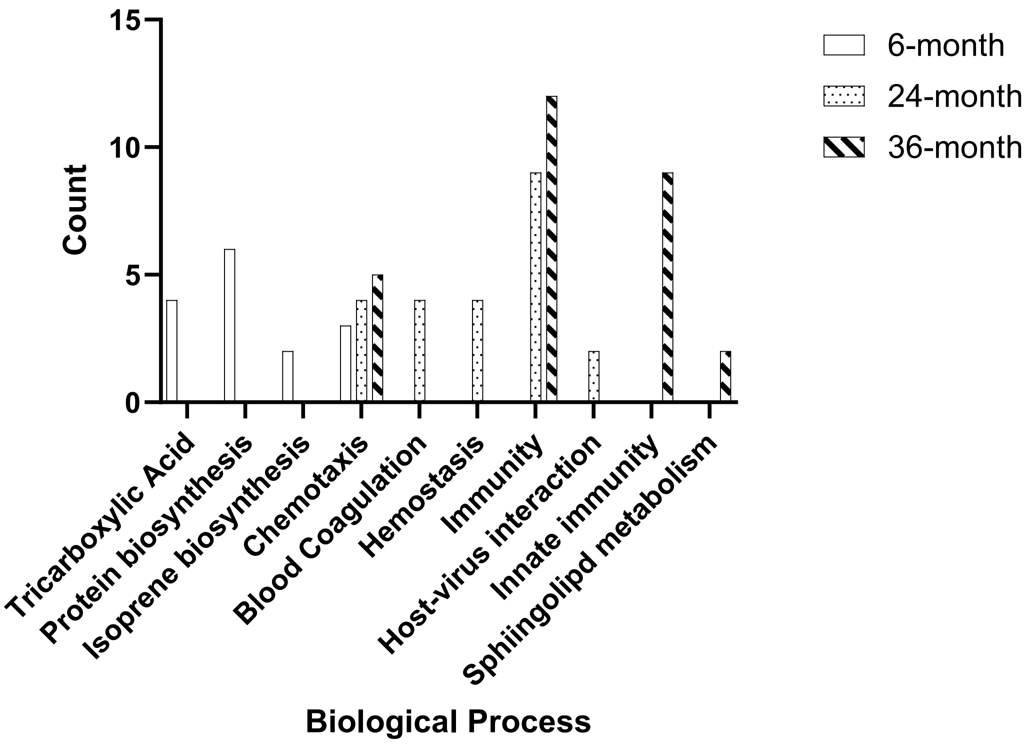


**Supplementary Figure 9:** Biological processes associated with genotype at 6-, 24-, and 36-month using the DAVID functional annotation tool. N= 6,9,9, respectively.

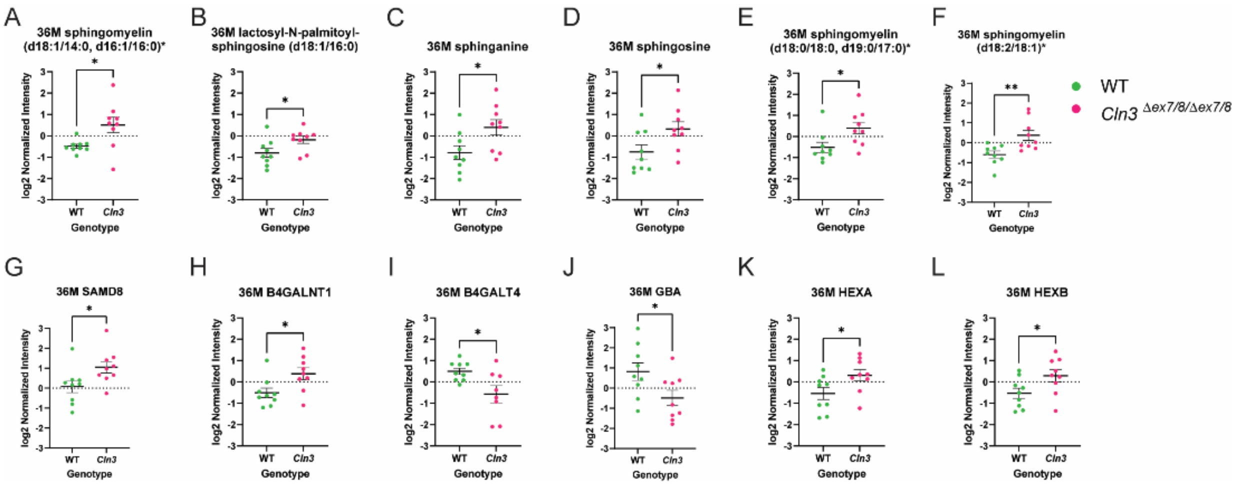


**Supplementary Figure 10: Metabolites and Proteins Involved in Sphingolipid Metabolism.** Metabolites and proteins involved in sphingolipid metabolism were compared in wildtype and *CLN3*$^{\Delta ex7/8}$ minipig serum samples at 36-months. (A-F) Differentially expressed metabolites at 36-months included sphingomyelin (d18:1/14:0, d16:1/16:0)*, lactosyl-N-palmitoyl-sphingosine (d18:1/16:0), sphinganine, sphingosine, sphingomyelin (d18:0/18:0, d19:0/17:0)*, and sphingomyelin (d18:2/18:1)*, all of which were upregulated in *CLN3*$^{\Delta ex7/8}$ serum samples. (G-L) Sphingolipid proteins SAMD8, B4GALNT1, HEXA, and HEXB were significantly upregulated in *CLN3*$^{\Delta ex7/8}$ minipigs, while B4GALT4 and GBA were significantly downregulated. Student's two-tailed t-test. N = 6; 9; 9 animals respectively. Exact p-values and source data can be found in Supplementary Data 10. All abbreviated protein names reflect official gene names under Human Genome Organization (HUGO).

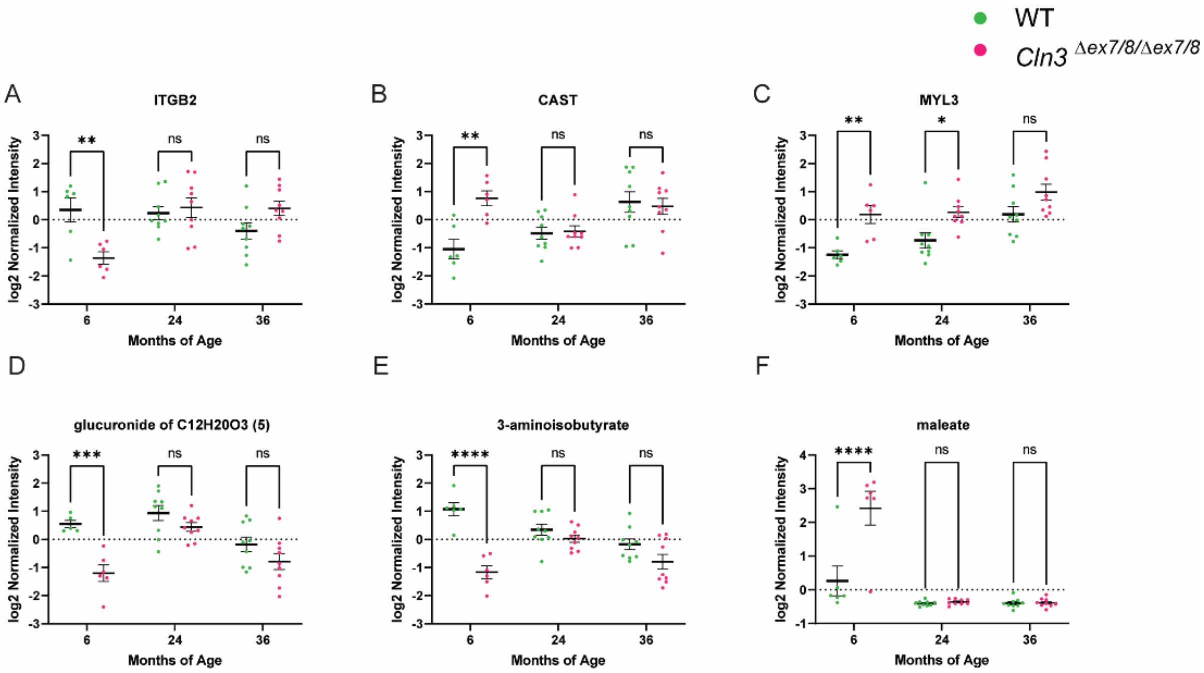


**Supplementary Figure 11: Identification of Biomarkers Appearing Early in Disease Progression, Prior to the Onset of Symptoms.** (A-C) Of the 230 protein targets associated with genotype at 6-months, Integrin Beta-2 (ITGB2), Calpastatin (CAST), and Myosin Light Chain 3 (MYL3) demonstrate significant dysregulation, all stabilizing to wild type levels at later time points. (D-F) Similarly, Glucuronide of $C_{12}H_2OO_3$, 3-aminoisobutyrate, and maleate demonstrate utility as "early" metabolite biomarkers and are only dysregulated at 6-months. Two-way ANOVA with Šidák correction for multiple comparisons, 95% confidence interval, n = 6; 9; 9 animals respectively, mean +/- SEM. Exact p-values and source data can be found in Supplementary Data 10.

**Supplementary Table 1:** The six potential Batten disease protein biomarkers identified by our pig plasma model were cross-referenced with previously reported plasma proteome data obtained through standard plasma proteomics workflows[15]. These workflows encompassed various approaches: "Neat" denotes a neat plasma digestion workflow, “Depleted" signifies the use of a plasma depletion strategy, “Deep Fractionation" involves a high-pH fractionation of depleted plasma, achieved by concatenating 19 fractions into 9, “Proteograph" represents a comprehensive five-NP workflow. In all these workflows, Data-Independent Acquisition (DIA) was utilized to analyze a pooled plasma sample. Notably, our pig plasma model demonstrated its capability to quantify novel putative disease biomarkers that are often undetectable using standard plasma proteomics workflows.

| | Neat | Depleted | Deep Fractionation | Proteograph | Pig plasma |
|---|---|---|---|---|---|
| **CTSB** | | X | X | X | X |
| **MHY7** | | | | X | X |
| **CTSS** | | X | X | X | X |
| **NFL** | | | | | |
| **MYL2** | | | | | X |
| **IFI30** | | | | | X |
