# Supplementary material for: Longitudinal deep multi-omics profiling in a CLN3Δex7/8 minipig model identifies biomarker signatures of disease
Source: Commun Med (Lond). 2026 Mar 3;6:132. doi: 10.1038/s43856-025-01227-5 (PMC12957377; doi:10.1038/s43856-025-01227-5)
Supplement: Supplementary file 3 — Description of additional supplementary files [file 43856_2025_1227_MOESM3_ESM.docx]

**Supplementary Data 1:** Source data for the Uniform Manifold Approximation and Projection (UMAP) of metabolites and proteins in Figure 2.

**Supplementary Data 2:** Formula used to calculate sPLS score from normalized data. These formulas were used to generate Figure 5 c, and d.

**Supplementary Data 3:** Contains the p-values and fold change of detected metabolites/lipids via RP/UPLC-MS/MS and HILIC/UPLC-MS/MS at 6-, 24-, and 36-months and is the source data for Figure 4, and Supplementary Figures 4 and 5.

**Supplementary Data 4:** Contains the p-values and fold changes of detected proteins via LC-MS/MS at 6-. 24-. And 36-months and is the source data for Figure 4, and Supplementary Figures 4 and 5.

**Supplementary Data 5:** Normalized metabolite/lipid concentrations measured via RP/UPLC-MS/MS and HILIC/UPLC-MS/MS in individual animals at 6-, 24-, and 36-months. Contains the source data for Figure 3, and Supplementary Figures 10 and 11.

**Supplementary Data 6:** Normalized protein concentrations measured via LC-MS/MS in individual animals at 6-, 24-, and 36-months. Contains the source data for Figure 3, and Supplementary Figures 10 and 11.

**Supplementary Data 7:** Bayesian analysis and corresponding p-values in 6-month samples. Contains the source data for Supplementary Figure 5.

**Supplementary Data 8:** Bayesian analysis and corresponding p-values in 24-month samples. Contains the source data for Supplementary Figure 6.

**Supplementary Data 9:** Bayesian analysis and corresponding p-values in 36-month samples. Contains the source data for Supplementary Figure 7.

**Supplementary Data 10:** Exact p-values for statistical comparisons made in Figure 3b-g. Figure 5c-d, and Supplementary Figure 10a-l, and Supplementary Figure 11a-f.

**Supplementary Data 11:** Contains numerical values for training set and held-out test set for each component of the sPLS score, as well as the sPLS score. Corresponding values are plotted in Figure 5d-f.
