# Supplementary material for: Longitudinal deep multi-omics profiling in a CLN3Δex7/8 minipig model identifies biomarker signatures of disease
Source: Commun Med (Lond). 2026 Mar 3;6:132. doi: 10.1038/s43856-025-01227-5 (PMC12957377; doi:10.1038/s43856-025-01227-5)
Supplement: Supplementary file 5 — Supplementary Data 2 [file 43856_2025_1227_MOESM5_ESM.pdf]

Supplementary Information:

Formula for calculating sPLS score from normalized data:

First protein component: $t_1^{(P)}$ = 0.983*CTSS + 0.236*CTSB - 0.096

First metabolite component: $t_1^{(M)}$ = 0.355*GPE + 0.864*GPI – 0.121

sPLS score = $t_1^{(M)} + t_1^{(P)}$ = 0.355*GPE + 0.864*GPI + 0.983*CTSS + 0.236*CTSB – 0.217
