# Supplementary material for: Longitudinal deep multi-omics profiling in a CLN3Δex7/8 minipig model identifies biomarker signatures of disease
Source: Commun Med (Lond). 2026 Mar 3;6:132. doi: 10.1038/s43856-025-01227-5 (PMC12957377; doi:10.1038/s43856-025-01227-5)
Supplement: Supplementary file 15 — Reporting Summary [file 43856_2025_1227_MOESM15_ESM.pdf]

nature portfolio

Corresponding author(s): Jon J. Brudvig

Last updated by author(s): Aug 15, 2025

Reporting Summary

Nature Portfolio wishes to improve the reproducibility of the work that we publish. This form provides structure for consistency and transparency in reporting. For further information on Nature Portfolio policies, see our Editorial Policies and the Editorial Policy Checklist.

Statistics

For all statistical analyses, confirm that the following items are present in the figure legend, table legend, main text, or Methods section.

n/a	Confirmed	
☐	☒	The exact sample size (*n*) for each experimental group/condition, given as a discrete number and unit of measurement
☐	☒	A statement on whether measurements were taken from distinct samples or whether the same sample was measured repeatedly
☐	☒	The statistical test(s) used AND whether they are one- or two-sided<br>*Only common tests should be described solely by name; describe more complex techniques in the Methods section.*
☒	☐	A description of all covariates tested
☒	☐	A description of any assumptions or corrections, such as tests of normality and adjustment for multiple comparisons
☐	☒	A full description of the statistical parameters including central tendency (e.g. means) or other basic estimates (e.g. regression coefficient) AND variation (e.g. standard deviation) or associated estimates of uncertainty (e.g. confidence intervals)
☐	☒	For null hypothesis testing, the test statistic (e.g. *F*, *t*, *r*) with confidence intervals, effect sizes, degrees of freedom and *P* value noted<br>*Give P values as exact values whenever suitable.*
☒	☐	For Bayesian analysis, information on the choice of priors and Markov chain Monte Carlo settings
☒	☐	For hierarchical and complex designs, identification of the appropriate level for tests and full reporting of outcomes
☒	☐	Estimates of effect sizes (e.g. Cohen's *d*, Pearson's *r*), indicating how they were calculated

Our web collection on statistics for biologists contains articles on many of the points above.

Software and code

Policy information about availability of computer code

Data collection

Proteomics: Samples were processed by SP100 automation instrument with ProteographTM Assay Kt included in the Proteograph Product Suite (Seer, Inc.) using five distinctly functionalized nanoparticles (NPs).

Metabolomics: Metabolon's hardware and software systems were used for raw data extraction, peak identification, and quality control processing.

Data analysis

Proteomics: All mass spectra files were analyzed with SpectroMine software (Biognosys, version 2.7.210226.47784). The R package {MSstatsTMT} version 2.2.7 was used to log2-transform the peptide intensities, impute within-TMT mixture missing values using an accelerated failure model, perform global median normalization on the peptide data (equalizing the medians across all channels and MS runs), conduct fraction aggregation, and perform protein quantification. The sets of proteins associated with genotype at each time point were assessed for enrichment in GO terms using Database for Annotation, Visualization, and Integrated Discovery (DAVID). The final dataset was reduced to a 2-dimensional uniform manifold approximation and projection (UMAP) using the uwot package in R21. The UMAP was then clustered using hclust (stats package in R) and the distance provided by the UMAP into 9 clusters, where the number of clusters was decided by the elbow method. The proteins from each cluster were then tested for enrichment of Uniprot Keywords using the AnnoCrawler pipeline (https://github.com/DansenCode/AnnoCrawler)

Metabolomics: Metabolon's hardware and software systems were used for raw data extraction, peak identification, and quality control processing. The sets of metabolites associated with genotype at each time point were assessed for enrichment in chemical structure types using MetaboAnalyst.

Both: Multiblock sparse partial least squares discriminant analysis (sPLS-DA), also known as DIABLE (Dta Integration Analysis for Biomaker discovery using Latenet variable approaches for Omics studies), was performed on the process proteomics and metabolomics datasets using the {mixOmics} package in R.
Bayesian Analysis:
Analytes (proteins and metabolites) that differed in abundance between the wild type and CLN3Δex7-8 samples at each time point (36-months) were identified via two-tailed Student's t-test. Median imputation was performed for missing values. 152 proteins and 63 metabolites were used in the network analysis. The log2 fold change values for analytes of individual CLN3Δex7-8 animals as compared to average wild type values were discretized using Cyni Toolbox Equal Width/ Frequency Discretization with 20 intervals. The Bayesian – Hill Climbing inference algorithm with Bayesian Dirichlet Equivalent (BDe) metric was used with the max number of parents as 3, reverse edges; only nodes with edges were outputted. The hill-climbing algorithm with a local search built the causal Bayesian network. The algorithm began with only nodes and iterates through the possibilities of adding, removing, or reversing an edge until the highest probability as measured with the BDe metric score is found. This process is repeated until no improvement is found. The BDe metric does not require prior expert knowledge and is based on likelihood equivalence. Networks were organized using the Perfuse Force Directed layout; scores from the Bayesian Hill Climbing algorithm were used as weight and were evaluated by the Heuristic function (min edge weight:0, max weight:1, default:0.5, num of iterations:100, spring coefficient: 1E-4, spring length:75, node mass:5, force deterministic layouts). The displayed edge weights correspond the BDe metric score; higher scores resulted in thicker edges. Sub-networks were created by selecting edges with scores greater than 0.001. The connected nodes and those nodes neighbors (directed: incoming) were selected.

For manuscripts utilizing custom algorithms or software that are central to the research but not yet described in published literature, software must be made available to editors and reviewers. We strongly encourage code deposition in a community repository (e.g. GitHub). See the Nature Portfolio guidelines for submitting code & software for further information.

Data

Policy information about availability of data

All manuscripts must include a data availability statement. This statement should provide the following information, where applicable:

- Accession codes, unique identifiers, or web links for publicly available datasets
- A description of any restrictions on data availability
- For clinical datasets or third party data, please ensure that the statement adheres to our policy

The metabolomics data supporting Fig. 2, and Figs. 4-5, are publicly available in the MetaboLights data repository, as part of the study number MTBLS1107. The proteomics dataset supporting Figs. 2-5, are publicly available via the following hyperlink https://massive.ucsd.edu/ProteoSAFe/dataset.jsp?task=c0856cd9532f47cfa86554f54ce91870

Research involving human participants, their data, or biological material

Policy information about studies with human participants or human data. See also policy information about sex, gender (identity/presentation), and sexual orientation and race, ethnicity and racism.

Reporting on sex and gender	*Use the terms sex (biological attribute) and gender (shaped by social and cultural circumstances) carefully in order to avoid confusing both terms. Indicate if findings apply to only one sex or gender; describe whether sex and gender were considered in study design; whether sex and/or gender was determined based on self-reporting or assigned and methods used. Provide in the source data disaggregated sex and gender data, where this information has been collected, and if consent has been obtained for sharing of individual-level data; provide overall numbers in this Reporting Summary. Please state if this information has not been collected. Report sex- and gender-based analyses where performed, justify reasons for lack of sex- and gender-based analysis.*
Reporting on race, ethnicity, or other socially relevant groupings	*Please specify the socially constructed or socially relevant categorization variable(s) used in your manuscript and explain why they were used. Please note that such variables should not be used as proxies for other socially constructed/relevant variables (for example, race or ethnicity should not be used as a proxy for socioeconomic status). Provide clear definitions of the relevant terms used, how they were provided (by the participants/respondents, the researchers, or third parties), and the method(s) used to classify people into the different categories (e.g. self-report, census or administrative data, social media data, etc.) Please provide details about how you controlled for confounding variables in your analyses.*
Population characteristics	*Describe the covariate-relevant population characteristics of the human research participants (e.g. age, genotypic information, past and current diagnosis and treatment categories). If you filled out the behavioural & social sciences study design questions and have nothing to add here, write "See above."*
Recruitment	*Describe how participants were recruited. Outline any potential self-selection bias or other biases that may be present and how these are likely to impact results.*
Ethics oversight	*Identify the organization(s) that approved the study protocol.*

Note that full information on the approval of the study protocol must also be provided in the manuscript.

Field-specific reporting

Please select the one below that is the best fit for your research. If you are not sure, read the appropriate sections before making your selection.

☒ Life sciences ☐ Behavioural & social sciences ☐ Ecological, evolutionary & environmental sciences

For a reference copy of the document with all sections, see nature.com/documents/nr-reporting-summary-flat.pdf

Life sciences study design

All studies must disclose on these points even when the disclosure is negative.

Sample size	Samples were collected as part of a pre-existing longitudinal phenotyping study (referenced in text). All available samples were used for analysis.
Data exclusions	48-month pig samples were excluded for reason discussed in the manuscript.
Replication	Rather than using a replication approach, models were built with a training set, while a held out test set was used for validation. No further samples were available for replication.
Randomization	All available samples were used. Samples were grouped by genotype with no randomization.
Blinding	Investigators were blinded to experimental group throughout analysis.

Reporting for specific materials, systems and methods

We require information from authors about some types of materials, experimental systems and methods used in many studies. Here, indicate whether each material, system or method listed is relevant to your study. If you are not sure if a list item applies to your research, read the appropriate section before selecting a response.

Materials & experimental systems

n/a	Involved in the study	
☒	☐	Antibodies
☒	☐	Eukaryotic cell lines
☒	☐	Palaeontology and archaeology
☐	☒	Animals and other organisms
☒	☐	Clinical data
☒	☐	Dual use research of concern
☒	☐	Plants

Methods

n/a	Involved in the study	
☒	☐	ChIP-seq
☒	☐	Flow cytometry
☒	☐	MRI-based neuroimaging

Animals and other research organisms

Policy information about studies involving animals; ARRIVE guidelines recommended for reporting animal research, and Sex and Gender in Research

Laboratory animals	Species: Sus scrofa<br>Strain: Yucatan miniature pig (Transgenic CLN3Δex7-8)<br>Age: Samples were collected at 6-months ± 7 weeks, 24-months ±10 weeks, 36-months ± 6 weeks, and 48-months ± 12 weeks in both CLN3Δex7-8 and control mini pigs
Wild animals	The study did not involve wild animals.
Reporting on sex	Sex-based analyses were not performed. As previously stated, sample numbers were limited and the study was not properly powered for sex-based analyses.
Field-collected samples	The study did not involve samples collected from the field.
Ethics oversight	Yucatan miniature pigs were housed and maintained at Exemplar Genetics under an approved Institutional Animal Care and Use Committee (IACUC) protocol.

Note that full information on the approval of the study protocol must also be provided in the manuscript.

Plants

Seed stocks	*Report on the source of all seed stocks or other plant material used. If applicable, state the seed stock centre and catalogue number. If plant specimens were collected from the field, describe the collection location, date and sampling procedures.*
Novel plant genotypes	*Describe the methods by which all novel plant genotypes were produced. This includes those generated by transgenic approaches, gene editing, chemical/radiation-based mutagenesis and hybridization. For transgenic lines, describe the transformation method, the number of independent lines analyzed and the generation upon which experiments were performed. For gene-edited lines, describe the editor used, the endogenous sequence targeted for editing, the targeting guide RNA sequence (if applicable) and how the editor was applied.*
Authentication	*Describe any authentication procedures for each seed stock used or novel genotype generated. Describe any experiments used to assess the effect of a mutation and, where applicable, how potential secondary effects (e.g. second site T-DNA insertions, mosiacism, off-target gene editing) were examined.*
